# Myasthenia gravis: a clinical-immunological update

**DOI:** 10.1007/s00415-015-7963-5

**Published:** 2015-12-24

**Authors:** Sophie Binks, Angela Vincent, Jacqueline Palace

**Affiliations:** Nuffield Department of Clinical Neurosciences, University of Oxford, John Radcliffe Hospital, Oxford, OX3 9DU UK

**Keywords:** Myasthenia gravis, MuSK, LRP4, IgG4, Cell-based assays, Neuromuscular junction, Thymectomy

## Abstract

Myasthenia gravis (MG) is the archetypic disorder of both the neuromuscular junction and autoantibody-mediated disease. In most patients, IgG1-dominant antibodies to acetylcholine receptors cause fatigable weakness of skeletal muscles. In the rest, a variable proportion possesses antibodies to muscle-specific tyrosine kinase while the remainder of seronegative MG is being explained through cell-based assays using a receptor-clustering technique and, to a lesser extent, proposed new antigenic targets. The incidence and prevalence of MG are increasing, particularly in the elderly. New treatments are being developed, and results from the randomised controlled trial of thymectomy in non-thymomatous MG, due for release in early 2016, will be of particular clinical value. To help navigate an evidence base of varying quality, practising clinicians may consult new MG guidelines in the fields of pregnancy, ocular and generalised MG (GMG). This review focuses on updates in epidemiology, immunology, therapeutic and clinical aspects of GMG in adults.

## Introduction

Myasthenia gravis (MG) represents the archetypic disorder of both the neuromuscular junction (NMJ) and autoantibody-mediated disease. In most patients, IgG1-dominant antibodies to acetylcholine receptors (AChRs) cause fatigable weakness of skeletal muscles with an ocular onset in up to 85 % [1]. A variable proportion of patients lacking AChR antibodies, termed seronegative MG (SNMG), possess antibodies to muscle-specific tyrosine kinase (MuSK) [2, 3] and intriguingly, these antibodies are principally IgG4 [3–5]. The remainder of SNMG is now rapidly being explained via cell-based assays (CBAs) using a receptor-clustering technique [6–8], and, to a lesser extent, proposed new antigenic targets [9].

The incidence and prevalence of MG are increasing, particularly in older individuals [10, 11]. However, MG remains a rare disease and there are well-documented impediments to clinical trials including low participant recruitment [12]. Indeed, the EPITOME trial [13] in ocular MG (OMG) had to close recently due to failure to recruit adequate numbers [14]. Nevertheless, rituximab appears to show promise in MuSK MG [15] and a much-anticipated randomised controlled trial (RCT) of thymectomy in non-thymomatous MG [16] is due to report in early 2016. These results will be of great value since thymectomy has been offered for many years in this setting, without incontrovertible evidence of benefit compared to purely medical management [17, 18].

Expert clinical guidelines have reviewed pregnancy in MG [19], and management guidelines have been published for OMG [20] and generalised MG (GMG) (with some comments on OMG) [21]. This review will focus on GMG, as recent updates on congenital myasthenia [22] and OMG [23] have already been published. However, in addition to the epidemiology, immunology, therapeutics and clinical management of GMG, ongoing efforts to define the risk of generalisation (ROG) from ocular to generalised MG will be described.

## Epidemiology: the changing face of myasthenia gravis

Calculations of total MG incidence and prevalence, based on 55 studies spanning 1950–2007, have yielded a pooled incidence rate (IR) of 5.3 per million person-years and a prevalence rate (PR) of 77.7 cases per million of the population [10]. Marked heterogeneity and the varying quality of epidemiological studies, were, not surprisingly, notable factors influencing these estimations over so many years [10]. Nevertheless, it is well recognised that MG prevalence has been rising since the middle of the last century [24], with improved recognition and diagnosis, medical and intensive care advances and patient longevity all playing a role [1, 10, 24].

The yearly incidence has also risen in all studies performed more recently [24, 25], due to a pronounced increase among older males as well as females [25, 26]. It remains appreciable even after adjustment for life expectancy [11, 27–29] and is not paralleled in younger females or children [30]. Studies of late-onset MG (LOMG) are hampered by the lack of unanimously agreed age of onset, with suggested cut-off points ranging from 40 to 75 years [1, 26, 28, 31–34] (see Box 1). The different HLA haplotype association in LOMG patients has been recognised since the 1980s [35], but the increase in incidence could also be related to environmental aspects [36] and better case detection [28].

**Box 1:** Features of LOMG in selected literature [1, 25, 26, 28, 31–34]

Authors	Country	LOMG prevalence	Onset age defined as
Evoli et al. [33]	Italy	20.5 % (172/837) of an MG clinic cohort	>60
Poulas et al. [25]	Greece	Point prevalence 175.37 per million population in ≥70 s, the highest of all age groups studied (range 4.7–175.37)	
Vincent et al. [26]	UK	Incidence rising to 9.9/100,000 per year in males and 4.8/100,000 in females	≥60
Meriggioli et al. [1]	N/a	N/a	≥40
Murai et al. [28]	Japan	LOMG/EOMG = 28.8 % of MG in 1987 vs. 41.7 % of MG in 2006 in a national epidemiological study	≥50^a^ (LOMG) ≥65^a^ (EOMG)
Živković et al. [34]	USA	66 % (114/174) of an MG clinic cohort	>50
Alkhawajah et al. [31]	Canada	>50 % MG, based on a prior regional epidemiological study [11]	≥65
De Meel et al. [32]	The Netherlands	35 % (34/96) of a University hospital MG cohort	≥50

^a^This study sub-divided patients into LOMG defined as ≥50 and elderly onset defined as ≥65

Described immunological changes that occur with ageing including diminished B and T cell repertoires and activation, but environmental factors are also implicated [36]. Although some investigators have reported a higher rate of thymomas in LOMG [28], thymic hyperplasia is less common in older individuals [31–33, 37] and thymectomy unusual unless thymoma is present, limiting samples available for study [1]. The advent of robotic and other minimally invasive operative techniques may alter this scenario, since data now suggest that the operation is safe in older individuals and potentially beneficial if hyperplasia is present [37, 38]. Whether the surgical fitness of these elderly Japanese cohorts can be extrapolated to other elderly populations requires consideration.

Currently, a UK multicentre trial to define immunological, phenotypic and clinical features, including optimal treatment, of LOMG is recruiting and at the time of writing is at 41 % of target [39]. This is important as, historically, there is evidence of misdiagnosis among older individuals, particularly with cerebrovascular disease [26] and new diagnoses have been made in those as old as 98 [40]. Older patients are less likely to enter complete stable remission [27, 31] and are more likely to suffer exacerbation with poorer outcomes, including death [32, 41]. Their management is also challenging because they are more likely to have co-morbidities [33] and are at higher risk of side-effects from acetylcholinesterase inhibition [42] and steroids [33].

### The epidemiological story in MuSK

MuSK MG has a younger age of onset and female predominance [43–47]. Initially, MuSK antibodies were reported to be prevalent in 70 % of ‘seronegative’ sera [2]. Subsequent cohorts, probably including patients with less severe disease, detected MuSK positivity less frequently in SNMG, but with wide variations [48]. For example, 3.8 % of SNMG tested positive for MuSK in a Chinese cohort [49] whereas in Italy the rate reached 47.4 % [44]. Accordingly, incidence and prevalence rates in MuSK MG epidemiological studies have differed with higher rates in Greece [annual IR of 0.32 patients/million population per year and prevalence rates (PR) of 2.92 per million population] compared to The Netherlands (where these rates are 0.10 and 1.9, respectively) [50, 51]. The variation in incidence in these studies and others [48; A Vincent, unpublished observations] support an environmental factor, but since there is also an association with HLA DQ5 across Dutch, Italian and Turkish populations [52–54], it suggests this acts on patients with a genetic predisposition.

## Immunological advances: new techniques, an emerging pathogenic player and proposed new antigenic targets

### Cell-based assays using clustered acetylcholine receptors

Traditionally, radioimmunoassays (RIA) (where the antigen is present in solution) are used for antibody ascertainment in MG. However, AChRs expressed on a mammalian cell line (human embryonic kidney cells, HEK) more closely mirror physiological conditions and increase sensitivity and specificity compared to solid phase assays for many antigenic targets [55, 56]. For MG, a clustered AChR cell-based assay (CBA) was established, in which the AChR subunits are expressed together with rapsyn, a post-synaptic protein crucial for AChR clustering at the NMJ. Using this method, AChR antibodies could be identified in 66 % of previously SNMG sera. These antibodies were primarily of the IgG1 sub-type and, in agreement with their pathogenic potential, could activate complement [6] and transfer neuromuscular transmission defects to mice [7].

50 % of previously seronegative OMG patients also had IgG1 clustered AChR antibodies [7]. As in all similar studies, it is not always clear whether the cohorts used are representative of the spectrum of incident cases, but clustered CBA antibodies have been found helpful in the diagnosis of young or childhood MG with often mild or ocular disease, responding well to immunotherapies [8]. The phenotype may become better defined as more laboratories adopt the clustered CBA [57].

The use of CBAs, with clustered antigens if appropriate, should improve both diagnosis and management of previously seronegative patients. Preliminary results from Oxford suggest 8 % of patients without MuSK or other antibodies bound detectably to MuSK expressed on HEK cells [56]. An international study probing seronegative sera from 13 countries for binding to MuSK by CBA identified antibodies in 13 % of samples [58], but many of the antibodies detected were predominantly of the IgM type, which is of unknown relevance.

### IgG4: a new pathogenic player

MuSK is a post-synaptic protein which is critical for the development and maintenance of the NMJ [reviewed in 59]. Agrin is released from the presynaptic nerve terminal and binds to low-density lipoprotein receptor 4 (Lrp4), which in turn binds to and activates MuSK. This leads to MuSK phosphorylation, ultimately resulting in the clustering of rapsyn and AChR on top of post-synaptic folds; this AChR clustering is essential for efficient transmission at the NMJ [60, 61].

MuSK antibodies are mainly IgG4 [3], in contrast to the IgG1 and IgG3 dominance of AChR antibodies. AChR antibodies principally act through complement activation, and by cross-linking and internalisation of receptors [1], both of which require antibody divalency. However, MuSK IgG4 antibodies are thought to be single chain rather than divalent, and do not activate complement or cause cross-linking of the MuSK molecules [4, 62]. Nevertheless, the pathogenicity of MuSK IgG4 antibodies has been demonstrated and overall the titres in a patient relate well to disease severity with reductions on remission [63, 64]. Injection of purified IgG4 into experimental animals leads to defects in neuromuscular transmission with AChR loss [65] as does injection of purified IgG [66].

MuSK IgG4 antibodies act through direct inhibition of MuSK-Lrp4 binding, without receptor dimerization or endocytosis, whereas this was not found with the remaining IgG1–3 antibodies [4, 5]. However, both these IgG1–3 antibodies and IgG4 antibodies could disperse AChR clusters in a mouse muscle cell line [4], implying that IgG1–3 could play a role in the disease; it is possible that by binding divalently, and activating phosphorylation, they cause desensitisation of MuSK leading to loss of function by that mechanism.

IgG4 antibodies were previously little known in autoimmune disease and thought to occur as a benign phenomenon in conjunction with resolution of allergic reactions [4, 62]. However, they are recognised in other diseases, such as forms of pemphigus, and represent a field of growing interest [62].

### Continuing the search for new epitopes

Although antibodies detectable via clustered CBAs are likely to be diagnostic in the majority of currently ‘seronegative’ patients, the hunt continues for antibodies to other elements of the NMJ which may be disease causative in smaller sub-sets of patients.

#### LRP4

As the partner of MuSK, Lrp4 is similarly essential in development and for normal function of the adult NMJ, where it performs both anterograde and retrograde signalling roles [67]. These roles highlighted it as a putative antigen of interest, and LRP4 antibodies have been reported in Japanese [68] and European [69, 70] patients. The antibodies were of the complement-activating IgG1 type [68] and impeded agrin-induced clustering of AChRs [69, 70]. These antibodies have now been examined by a number of groups and overall their presence in seronegative sera has varied widely (Table 1).

**Table 1:** Prevalence of LRP4 antibodies in studies of seronegative MG [68–71]

Investigators	Experimental method	Prevalence in SNMG	Definition of SNMG	Double positives
Higuchi et al. [68]	Luciferase-reporter immunoprecipitation	3 % (9/300)	AChR −ve	3 of the 9 LRP4 +ve samples were also MuSK +ve
Pevzner et al. [69]	CBA	54 % (7/13)	AChR and MuSK −ve	A control MuSK sample was also LRP4 +ve
Zhang et al. [70]	ELISA	9.2 % (11/120)	AChR and MuSK −ve	1 of 36 MuSK samples tested was also LRP4 +ve
Zisimopoulou et al. [71]	CBA	18.7 % (119/635)	AChR and MuSK −ve	8/107 AChR +ve and 10/67 MuSK +ve samples also LRP4 +ve

These discrepant results are likely to emanate at least in part from the different assays used by separate research groups [9]. The potential of certain commercial secondary antibodies to detect non-specific binding of IgM antibodies to MuSK has been noted (S Huda, unpublished results). In addition to clarifying the specificities and sensitivities of these different LRP4 antibody assays, the relevance of the antibodies when found in association with other pathogenic myasthenia antibodies (see Table 1) needs exploration. Further definition of the clinical phenotype of LRP4 disease is also required although present information suggests this is a predominantly female cohort with mild symptoms, similar to AChR-antibody-positive patients [69, 71].

#### Agrin and ColQ

Antibodies to agrin have recently been identified in a small number of ‘triple negative’ MG sera (samples negative for AChR, MuSK and LRP4 antibodies) at proportions ranging from 15 to 50 % [9, 72]. These antibodies have sometimes been at low titres [72] and can be found with [9, 72] or only with AChR or MuSK antibodies [73]. This suggests technical difficulties and methodologies need to improve before the significance of agrin antibodies can be evaluated.

ColQ tethers MuSK within the synapse [74] and is thought to interact also with MuSK. ColQ antibodies were reported in 3–4 % of all MG patient sera tested and 1.2–5.5 % of the AChR/MuSK/LRP4 negative samples [9, 75] but again the specificities are unclear and further work is required to delineate their role [75].

## A link with antibody-mediated demyelinating disorders?

The co-occurrence of MG and demyelinating disorders happens more than would be expected by chance [76]. Recently, a cohort of 16 patients with neuromyelitis optica (NMO) and MG was described. In such cases, the MG tended to be mild and 90 % presented with a prior history of NMO; however, Aquaporin-4 (AQP4) antibodies could pre-date clinically evident NMO by up to 16 years [76]. Of note, in other case series of MG presenting with demyelinating disorders described as MS or ADEM, AQP4 testing was not reported; some of these may have represented NMO [77–79] and in at least one case this was subsequently confirmed [78]. LRP4 antibodies have also been described in NMO patients, albeit without a known diagnosis of MG, but not other neurological disorders [70]. Why these two diseases should occur in this order, with AQP4 antibodies rising over time, is not understood, although it is speculated that thymectomy may play a triggering role. Clinicians should at least be vigilant to the possible co-existence of NMO with MG, particularly in young patients with AChR-antibody-positive disease and should test for AQP4 antibodies in MG patients who develop MS or other demyelinating disorders [76].

## Risk of generalisation from OMG: setbacks and progress

The debate over the risk of generalisation from ocular to generalised MG continues. Retrospective patient data indicate that most OMG patients who develop GMG do so in the first 2 years [80–82]. However, unanimity has not been reached on other risk factors, or on whether early intervention with immunosuppressants, particularly corticosteroids, can delay or prevent the onset of GMG [23, 83]. This is of growing relevance in the era of expanding therapeutic and surgical options, and to avoid exposing patients to unnecessary adverse effects of immunotherapies.

Several retrospective cohorts have reported lower rates of generalisation in patients treated with corticosteroids compared to those treated with acetylcholinesterase inhibitors alone [81, 82, 84], but solid conclusions cannot be drawn from these non-randomised studies where confounding factors such as duration of OMG, serological status and differing steroid regimes are present. In some cases, patients received lengthy courses of steroids (up to 92 months) or were maintained on low-dose steroid regimes, either of which could have masked the development of GMG [82, 84]. Moreover, Grob’s survey of nearly 2000 patients between 1940 and 2000 found stable rates of generalisation from OMG [85], which might seem to contradict a disease-modifying effect of corticosteroid treatment.

The EPITOME trial [13], which was due to address these issues, would therefore have been of great clinical utility but unfortunately its closure was recently announced due to poor recruitment [14]. A UK initiative to develop a prognostic ‘ROG’ score is still in progress and reported preliminary results at the 2015 Association of British Neurologists conference. Using available case notes, investigators identified the three positive factors most predictive of secondary generalisation as being thymic hyperplasia, seropositivity and co-morbidity (including but not limited to other autoimmune disorders) [86]. Such a model should now be prospectively validated and could help identify high-risk patients for whom early immunosuppression would be beneficial.

### The therapeutic landscape

Pyridostigmine and corticosteroids retain a central role in the management of GMG [87]. Use of azathioprine as a steroid-sparing agent is supported by an RCT [88] but limited high quality evidence underlies many other immunosuppressants [87]. However, recently, a single-blinded trial proposed methotrexate as an alternative to azathioprine [89]. While this trial was devised to validate methotrexate in a resource-limited setting, it may have applicability for azathioprine-intolerant individuals.

MuSK MG patients have traditionally represented a clinical challenge as they exhibit poor response to acetylcholinesterase inhibitors [43–45, 90]. Rituximab, an anti-CD20 monoclonal antibody, is emerging as a potential option in this cohort [15, 91–93]. Following rituximab treatment, some patients even revert to a seronegative status [15]. Of particular interest, specific monitoring of IgG sub-classes in five clinically improved rituximab-treated MuSK MG individuals demonstrated significantly reduced IgG4 titres in all five. On the other hand, both clinical and serologic impact was much less favourable in AChR antibody patients treated in the same study [92]. Indeed, rituximab appears to be a useful treatment in other IgG4-related diseases and to act by eliminating a population of B- or plasma cells responsible for the production of IgG4 antibodies [62, 92]. Rituximab’s effect on T cell response may also be relevant, and an increase in T-regulatory cells has been observed post-rituximab administration in a refractory MuSK, but not AChR-positive, patient [94]. Further work is required to determine the optimal timing and administration schedule of rituximab in MG [93].

Another monoclonal antibody being considered for MG is eculizumab, which targets the C5 protein of the complement cascade, and so might protect the NMJ from complement-mediated damage. In a small phase II trial, there was significant change on the quantitative myasthenia gravis score (QMGS) with eculizumab compared to placebo [95]. A phase III trial is now in progress, aiming to enrol 92 patients [96]. The weekly dosing schedule and high cost of this medication may limit its use.

Early stage agents in development include EN101/Monarsen, an antisense oligonucleotide to mRNA of a splicing variant of acetylcholinesterase which is elevated in mice with experimental autoimmune MG and in patients. It is currently unclear whether clinical effect is due to inhibition of the splicing variant, producing symptomatic relief, or anti-inflammatory and immunomodulatory actions via the NF-κB pathway [97, 98]. Another drug in phase II studies is Tirasemtiv, which enhances skeletal muscle’s response to calcium and may be of benefit in combination with acetylcholinesterase inhibitors [99].

### Emergency treatments

A RCT of plasma exchange (PLEX) compared to IVIg in myasthenic crisis found equivalence between the two treatments [100, 101]. After 2 weeks, similar numbers improved in both groups, as measured on the QMGS. Although more patients (17.5 %) had worse 2-week QMGS scores in the IVIg group compared to those receiving PLEX (2 %), this was non-significant [100]. Recent hospital data show a trend to declining use of PLEX, which may be prompted by the invasive nature of this treatment modality [41] and its lack of availability in many centres. Nevertheless, it is important to note that in several cohorts, MuSK MG patients appear to respond less well to IVIg than PLEX [43, 45, 47, 90].

## Thymectomy: towards a definitive answer

The announcement of the results of the MGTX RCT of thymectomy vs. medical treatment in AChR-antibody-positive GMG patients will take place in Oxford in early 2016 and is likely to be a milestone event for myasthenic treatment. The protocol of this multicentre (>40 centre) trial has previously been published and is a single-blind, double armed trial evaluating trans-sternal thymectomy versus no operation in patients on prednisolone [16]. Some initial reports of thymic pathology in trial participants have been published and revealed 25–40 % thymic hyperplasia depending on the immunostaining method used, and parenchymal changes comparable to the non-MG population [102].

Where a thymoma is present, thymectomy is indicated to treat the tumour but not the myasthenia; however, frail and elderly patients are sometimes treated medically. On the other hand, in MuSK MG patients, thymic pathology is relatively rare [45, 90, 103, 104] although cannot be precluded [46, 105]. Few MuSK patients appear to improve following the procedure [43, 44] or, similar to AChR patients, are maintained on corticosteroids [47] which cloud the interpretation of any operative effects. Until MGTX reports, the situation remains difficult with no high quality evidence available to support decision making [16, 17]. The American Academy of Neurology (AAN) has developed guidelines to assist in this scenario [17], advising thymectomy be viewed as an option to improve clinical status and remission rates.

Another conundrum is whether a trans-sternal, minimally invasive or robotic approach offers best results. The MGTX will not answer this question, but it is sensible first to establish clinical benefit of any operative intervention prior to probing competing techniques [106]. Comparable results of around 28–34 % complete stable remission (CSR) or CSR and pharmacological remission (PR) have been achieved in single-centre, non-randomised case series from different hospitals [106–110]. One single-centre review of patient records displayed superior rates of complete remission with robotic (39.25 %) compared to thoracoscopic surgery (20.3 %), but the dates of all thoracoscopic surgeries predated robotic procedures, introducing the possibility of confounding historical factors [111].

## New best practice guidelines

New best practice guidelines have been released in the past 2 years which address ocular and generalised MG as well as pregnancy in MG [19–21]. Key points are summarised in Box 2. An important message for expectant mothers is that birth plans should aim for a hospital delivery as babies are at risk of transient neonatal MG irrespective of the mother’s disease status. Therefore, home births and midwife-led units are not advised [21].

**Box 2:** Take-home messages from recent best practice guidelines [19–21]

Myasthenia in pregnancy: best practice guidelines from a UK multispecialty working group
Norwood et al. [19]
Key points
Importance of pre-conception planning
Pyridostigmine, prednisolone (at lowest dose) and azathioprine may be used
Mycophenolate and methotrexate are teratogenic and contra-indicated in pregnancy
Monitoring, e.g. gestational diabetes in women on steroids
Aim for a vaginal delivery, but supported by multidisciplinary expertise
Babies need post-delivery monitoring due to risk of transient neonatal MG
Home births and midwife-led units are therefore not recommended
EFNS/ENS Guidelines for the treatment of ocular myasthenia
Kerty et al. [20]
Key points
Start with pyridostigmine treatment
Add in steroids if symptoms not controlled (will be required in most cases)
Next line is azathioprine
Some reports suggest thymectomy may reduce risk of secondary generalisation
Myasthenia: association of British Neurologists’ management guidelines
Sussman et al. [21]
Key points
First line tests: AChR antibodies, thyroid function tests, thymus scan
Second line tests: MuSK antibodies, neurophysiology, MRI brain
Provides escalation protocols for pyridostigmine and steroids in both OMG and GMG, including the option of every other day dosing
Advises bone protection
Azathioprine is first line in patients who do not achieve remission on prednisolone or who require long-term steroid doses in excess of 15-20 mg on alternate days
IVIg or PLEX may be given in crisis (PLEX if specific risk factors)
Thymectomy should be performed in a specialist centre with an experienced surgeon
Thymectomy in non-thymomatous MG is a ‘reasonable treatment option’ for patients <45 who are AChR antibody positive

## Conclusions

This paper has reviewed a number of evolving areas in GMG. In particular, patient diagnosis and management will improve as the pool of ‘seronegative’ MG decreases. However, care should be taken to establish the pathogenicity of newly identified antibodies. It is likely the field of IgG4-mediated disease will continue to gain scientific momentum.

More work is required to understand the phenomenon of increasing incidence of LOMG, with elderly patients posing a diagnostic and therapeutic challenge. Within the next 12 months, the results of the MGTX trial may answer one of the longest-standing questions in MG, namely, the role of thymectomy in non-thymomatous disease.
